# Glucocorticosteroid in Treatment of Severe Pneumonia

**DOI:** 10.1155/2013/865635

**Published:** 2013-12-02

**Authors:** Felinda Ariani, Kaixiong Liu, Zhang Jing, Jieming Qu

**Affiliations:** ^1^Department of Respiratory Medicine, Huadong Hospital, Shanghai Medical School of Fudan University, 221 Yananxi Road, Shanghai 200040, China; ^2^Department of Respiratory Disease, The First Affiliated Hospital, Fujian Medical University, 20 Chazhong Road, Fuzhou 350005, China; ^3^Department of Pulmonary Medicine, Zhongshan Hospital, Shanghai Medical School of Fudan University, 180 Fenglin Road, Shanghai 200032, China

## Abstract

Airway diseases such as pneumonia constitute a major health burden on a global scale; untreated pneumonia may develop to severe pneumonia and consequently lead to to fatal episodes of mortality and morbidity. The balance between inflammatory mediators is key for the outcome of the pulmonary infection; elimination of invading pathogen was marked by the release of cytokines and other inflammatory mediators from alveolar macrophages and glucocorticoid steroids (GCs) acting on the inflammatory component. Treatments of severe pneumonia with GCs have been developing for years with inconclusive results. In many cases GCs have been administered empirically without clinical evidence. Recent studies assess beneficial impact on treatment of severe pneumonia by suggesting specific dosage, period of administration, and tapered dosage.

## 1. Background

Severe pneumonia patients represent a major concern for physicians because of the high mortality and morbidity rate attributed to these episodes [1]. During past decades, many strategies have been implemented with the aim of optimizing the outcome of patients with severe lung infections. State of immunocompromisation during severe pneumonia related to multiple drug-resistant infections which may contribute to severe hypoxemic respiratory failure triggering septic shock and fatal outcome associated with multiple organ dysfunction syndromes. Not only is colonizing of bacteria responsible as main coordinators but it is believed that excessive inflammatory cascade is also responsible in the core of immune reaction. Nowadays, antimicrobial therapy not completely enough to significantly reduce mortality number in severe pneumonia, additional therapy such GCs may constitute an important portion for better resolution of pneumonia. If not treated properly, severe pneumonia can eventually lead to several complications including acute respiratory distress syndrome (ARDS) and sepsis. They are characterized by persistent pulmonary inflammation and alveolar-capillary disruption and commonly affect critically ill patients, with an estimated mortality rate of more than 50% [2]. We reviewed recent reports to clarify whether systemic corticosteroids have an impact on the outcomes of patients with severe pneumonia. In addition, we explored possible explanations for the role mechanism of corticosteroid in severe pneumonia.

## 2. Role of Glucocorticosteroid in Sepsis and ARDS

ARDS is common and frequently fatal; two pathological feature of lung derived from pulmonary fibrosis and sepsis, secondary to pneumonia, are the primary etiology of death in patients with late ARDS (>3 days). Cytokine levels TNF, IL-6 and IL-10 were highest in CAP (82%) with fatal severe sepsis and lowest in CAP with no severe sepsis [3].

For many decades, many studies have been observing implication of GCs in shock or sepsis to reduce mortality; nevertheless, it deals with inconclusive results. At the beginning, it was accepted to administer high-dose steroids, using either methylprednisolone (30 mg/kg) or dexamethasone (3–6 mg/kg), in divided doses for 1 to 2 days to treat patients with severe sepsis and septic shock [4–6], yet later, high-dose GCs showed possible harm and failed to decrease mortality [7]. After all, the enthusiasm to evaluate efficacy of GCs still became a topic.

However, low doses of GCs successfully decreased mortality rates [8, 9]. Several meta-analyses confirmed the survival and hemodynamic benefit concerning the use of low-dose hydrocortisone [10–12]. Earlier clinical study of low-dose methylprednisolone (with a loading dose of 1-2 mg/kg followed by 2 mg/kg per day) at an early phase of postoperative ARDS showed suppression in fibroproliferation as an early state response to lung injury and decreased CRP [13, 14]. Improved outcome, such as significant reduction of cytokine in plasma and BALF, improved oxygenation index, decreased lung injury score, and MODS also stated benefit GCs in late ARDS [15]. In contrast, high-dose steroid therapies were associated with increased mortality [16].

Eventually, new recommendation surviving sepsis campaign (SSC) protocol was developed as an international assessment to reduce mortality due to septic shock. It summarized a few key points: a stress-dose GCs therapy given only in septic shock after blood pressure was identified to be poorly responsive to fluid and vasopressor therapy. High doses of GCs comparable to >300 mg hydrocortisone daily cannot be administered in severe sepsis or septic shock. It also suggested that GCs cannot be administered for the treatment of sepsis in the absence of shock [17].

## 3. Cytokine Expression in Severe Pneumonia

Cytokine plays important role in sending signals cell to cell within immune response. The crucial role of inflammation in the lung will depend on expression complex group of proinflammatory mediator and cytokine response. Severities of pneumonia are closely related to elevated uncontrolled cytokine. One cohort investigated severe sepsis as CAP followed by organ dysfunction and mortality revealed cytokine level elevation happened in 82% of all subjects with CAP, whereas cytokine concentrations were highest at onset, attenuated rapidly over the first few days, but remained elevated throughout the first week. Activity level of the proinflammatory IL-6 and anti-inflammatory IL-10 cytokine significantly upraised prior to mortality [18].

Montón et al. studied demonstrated that patients who were receiving GCs, concentrations of TNF-*α*, IL-1*β*, IL-6, and CRP were remarkably decreased in serum and BAL (bronchoalveolar lavage) [19]. Recent studies also observed the production of IL-6, IL-17, IL-23, TNF-*α*, macrophage inflammatory protein-1a, monocyte chemotactic protein-1, keratinocyte-derived chemokine (KC), and interferon-g levels has superior suppression effects by the combination of clarithromycin and dexamethasone [20].

## 4. Mechanism Action of Glucocorticosteroid (GCs)

The anti-inflammatory and immuneosuppression process of GCs defined to two mechanisms: genomic mechanism and nongenomic mechanism, the prior means directly DNA binding (transactivation) hence inactivation transcription factor (transrepression) [21]. First, the ligand-activated GRa binds as a homodimer to Glucocorticosteroid responsive element (Gre) in target genes and induce transcription of DNA code, which is called transactivation. Second, cross-talk mechanism, as a regulation of gene expression in which GC-liganded GRs upregulated or downregulate inflammatory transcription factor proteins such as nuclear factor-*κ*B (NF-*κ*B) and activator protein-1 (AP-1), is defined as transrepression [22].

Another mechanism is GC signaling through membrane-associated receptors and second messengers described as nongenomic pathways. This mode of action entails mechanisms that do not directly and initially influence gene expression, and their effects are not blunted by inhibitors of gene transcription. Nongenomic mechanism involves the activation of endothelial nitric oxide synthetase (eNOS). Binding of GCs to the GR stimulates phosphatidylinositol-3′-kinase and Akt kinase, leading to eNOS activation and nitric oxide-dependent vasorelaxation. Nitric oxides participate in many of the inflammatory manifestations, including vasodilation and inflammatory cell recruitment [23, 24]. Dysregulation systemic inflammation stated with excessive production of proinflammatory transcription factor nuclear factor-kappaB (NF-*κ*B) and failing inhibitory action of anti-inflammatory transcription factor GCs receptor is central to the pathogenesis of pulmonary and extrapulmonary organ dysfunction within ARDS patients.

## 5. Critical Illness-Related Corticosteroid Insufficiency in Severe Pneumonia (CIRCI)

Despite their excellent anti-inflammatory efficacy, the use of GCs as therapeutics is often restrained due to two major drawbacks. First, long-term treatment with GCs is often accompanied by severe side effects, such as diabetes, increased risk of infection, osteoporosis, hypertension, and so forth [25, 26]. Occurrence of GCs resistance also restricts many GC-based therapies. Second reason, under normal condition hypothalamic-pituitary-adrenal (HPA) axis regulates the secretion GCs, suppression of HPA axis and adrenal failure may result in inadequate GCs activity to down regulate inflammatory response in severe illness patients. Few studies investigated the relationship between adrenal function and severity of pneumonia. Increased serum cortisol concentration was reported and shown to be linked with severity and mortality in CAP [27, 28].

Poor prognosis in ARDS patients is frequently associated with failure of the activated GRs to suppress the transcription of inflammatory cytokines, at the same time peripherally generated TNF-*α*, IL-1*β*, and IL-6 that can stimulate the HPA axis levels independently or synergistically thus indirectly suppress the immune response [29]. Alteration of GR-binding affinity also has been demonstrated in severe sepsis and septic shock [30].

As described by Annane et al., patients were classified as having CIRCI if baseline serum cortisol level is ≤10 *μ*g/dL or when the increase of serum cortisol after cosyntropin stimulation is ≤9 *μ*g/dL. They also suggested that the metyrapone test might be a useful tool for diagnosis of CIRCI [31]. Currently, the Surviving Sepsis Campaign recommends that the initially 200 mg/24 hours intravenous hydrocortisone therapy should be considered in septic shock if patients did not adequately respond to fluid resuscitation and vasopressor agents [17].

## 6. Role of Glucorticosteroid in Severe Pneumonia: Clinical Evidence

Severe pneumonia is one of the leading causes of Intensive Care Unit (ICU) admission among septic patients [32] with incidence of pneumonia increasing with age, septic shock, insufficient antibiotic treatment, respiratory failure, acute lung injury, and other complex factors related to this poor prognosis. The use of GCs has been debated over the past 50 years. Earlier in 1974, as pioneer, Weitzman and Berger reviewed the clinical trial design of studies reporting GCs use in bacterial infections [33]. At the beginning of the previous decade, high-dose GCs was generally accepted by practitioners. In 1995, meta-analyses found no benefit for high-dose GCs in sepsis and septic shock [34] and in the following years another meta-analyses found benefit for long duration of low-dose GCs [35].

Innovative treatments of GCs in severe pneumonia have emerged from septic shock field. Initially, Marik et al. investigated that there was no difference between placebo and low-dose hydrocortisone (10 mg/kg) group [36]; in contrast, role of high-dose GCs (mean ± SD dose of i.v. methylprednisolone 677 ± 508 mg for 9 ± 7 days) assessed by Montón et al. was demonstrating that GCs decreased systemic and lung inflammatory responses (IL-6, BAL neutrophilic count, and CRP) in mechanically ventilated patients [37]. Despite providing a benefit, this study lacks sample size and it should be noticed the using broad spectrum antibiotics may attenuate inflammatory response in alveolar cells and alleviate mechanical ventilation-induced lung injury as seen in vitro of mice experiment [38].

The use of GCs has been more consistently debated; however, the use of stress-dose or low-dose GCs for patients with septic shock improve hemodynamic function and provide survival benefit [39]. Hence, it become a turning point, low doses of GCs were administered successfully in preliminary sepsis complicated with severe CAP, it showed some benefits by decreasing mortality, mechanical ventilation use and length of stay. Although this study show beneficial effect of GCs, likewise previous study, small number of participant may biased efficacy of treatment [40]. These investigations rejuvenated enthusiasm to evaluate low-dose GCs treatment in patients with severe pneumonia. The confirmatory potential benefits, including survival advantage, reduced the number of organ system failures, the length of ICU duration, and mechanical ventilation [41–44], while others showed some benefit; two studies in end results were contradicted or even increased in accompanying adverse effects [45].

Snijder et al. in a study performed in 213 CAP hospitalized patients showed that 40 mg of prednisolone for 7 days or placebo, along with antibiotics, did not show difference in clinical outcome, no decline in CRP levels, or faster attenuated of fever and even lead to late failure. The result showed a more frequent hyperglycemia (2.3% versus 0.9% with *P* = 0.27), as well as superinfection occurred 2.1% versus 1.9% *P* = 0.10) and one patient in the placebo group developed a fungal infection after he was treated with hydrocortisone. The discordant results from Snijder et al. regarding inclusion of patients with nonsevere pneumonia resulted in a higher rate of adverse effects. Adverse effect may occur due to resistance to antibiotics or GCs itself [46].

The incidence of a rebound inflammation after initial suppression by GCs become an important issue [40, 42]. In this context, GCs inhibit cytokines and other inflammatory mediators accelerated by bacterial infections that can be harmful to the host. However, the use of GCs also brings a great influence in the immune function of macrophages and granulocytes as the main cell host defenses against bacteria [47]. Assumption of rebound inflammation is detected by higher CRP in prednisolone group after 2 weeks which was initially declined in the first week [45]. This likely indicates the fact that GCs do confer benefit in the earlier, whether by mechanism of cytokine suppression, activation of adrenergic, or amelioration of relative adrenal insufficiency, but precaution in their use should further investigate including susceptibility for infection.

As mentioned earlier, studies published in earlier 1990s were impressed with “mega” dose GCs using either methylprednisolone (30 mg/kg) or dexamethasone (3–6 mg/kg) divided into 2 days or single bolus dose [48]. Hereafter, studies obtained indication of harm and dramatically increasing mortality with high-dose GCs treatment for sepsis. Finally, low doses of GCs <300 mg/day were used successfully in sepsis studies [49, 50]. And nowadays, recommendations for the use of GCs in septic shock for administration are of 200–300 mg/day of hydrocortisone [17].

The underlying hypotheses about successful low-dose GCs at these doses attenuated some inflammatory responses [51]. Improved vasopressor responsiveness of the peripheral vessels [47], increased mean arterial pressures, and systemic vascular resistance [52] boost innate immunity in patients with septic shock [53] and coagulation disorders secondary to an infection [54]. These are main reasons why low-dose GCs were recommended in two large trials. Therefore, physicians can reverse shock and improve survival with GCs [55] as shown in the prospective, double-blind study reported by Keh et al. [49]. In fact, this kind of strategy could also work in severe CAP; however, the duration of therapy is likely to be 3 days to 2 weeks with a slow and progressive decreased in the dose. Study by Meijvis et al., GCs were administered once daily for 3 days [42], in addition in study by Yildiz et al. [39], GCs were administered with continuous infusion for 7 days; in addition in a study by Confalonieri et al. [40], GCs were administered approximately for 11.4 days. In contrast, analysis results of Salluh et al. [45] with 7days continuous or orally showed no improvement in clinical outcome; moreover, there was also indication of rebound inflammation.

## 7. Role of Glucorticosteroid in Specific Condition

Progressions of disease caused by respiratory viruses are often complicated by severe pneumonia. There is strong evidence that the interactions between pandemic strain and secondary bacterial respiratory pathogens are related to high incidence of ARDS and lung injury [56, 57]. Previous studies suggested that progression of disease to respiratory failure may be primarily mediated by host immune system despite decreasing viral replication [58]. Huge amounts of several proinflammatory cytokines are released in lung parenchyma [59]. Evidence suggested that corticosteroid use could achieve adequate control of excessive inflammation [60].

### 7.1. Influenza

Most of pandemic influenza A/H5N1 and part of seasonal and pandemic H1N1 patients result in deterioration outcome, they are at risk of quickly progressing to refractory hypoxemia-in need mechanical ventilation, thus frequently complicated with severe pneumonia, ARDS, and MODS. Treatment assessment of the use of GCs in human avian influenza is limited and lacked cases. GCs have been used clinically in the management of H5N1 patients associated with ARDS in Hong Kong, Vietnam, Indonesia, and Thailand; regarding confounding factors there was no evidence that showed a beneficial therapy of GCs in H5N1 patients [56, 61–63].

Later, few animal studies have been conducted regarding GCs use in avian influenza. The efficacy of dexamethasone, a potent and long-lasting glucocorticoid, was not responsive in inhibiting the development of acute respiratory syndrome associated with H5N1 virus-induced ARDS in mice [64].

Carter reviewed the articles and concluded that because of confounding factors and the fact that no large randomized clinical trials were conducted, it was not possible to gain any conclusions, but GCs may take effect if given at a low dose and administered for a sufficient time (7–10 days) [60].

Since the outbreak of H1N1, a minority of patients might lead to rapidly progressive pneumonia following acute lung injury (ALI)—acute respiratory distress syndrome (ARDS). GCs have often been setting as adjuvant therapy besides antiviral therapy and other measurements in an attempt to suppress the damage caused by the immune response.

Burn-Buisson et al. found that early course of GCs (median initial dose of 270 mg equivalent hydrocortisone per day for a median of 11 d) in patients with influenza A/H1N1 pneumonia associated with ARDS may be hazardous, with higher mortality and more likely to have superinfection [65]. Beneficial effect improvement in lung injury score, multiple organ dysfunction scores, and low mortality rate reported by Quispe Laime et al. prolonged low-to-moderate dose of GCs patients with severe ARDS received methylprednisolone (1 mg/kg/day), and others received hydrocortisone (300 mg/day) for a duration of 21 ± 6 days [66]. A better clinical outcome also provided by Kudo et al. is that GCs together with early administration of antiviral agents to pneumonia with wheezing and possibly without wheezing due to H1N1 may prevent patient's progression to severe pneumonia [67].

The addition of low dose of methylprednisolone infusion at a stress dose (1 mg/kg/24 h) as rescue therapy on 7 days also shows weaning from ECMO and invasive mechanical ventilation, and gradually reduce CRP levels and procalcitonin levels. Others 2 studies were associated with a significance beneficial therapy in severely ill patients which were unresponsive to other treatments, furthermore leading to rapid improvement with resolution of the pulmonary infiltrates and may decreased the viral load of H1N1 [57, 68, 69]. In contrary, studies by Liem et al. assumed treatment with methylprednisolone (1–3 mg/kg/day for up to 7 days) progressed to mortality (65% versus 29%; *P* = 0.004) [70]. Other studies suggest that there was no benefit in improving symptoms, but GCs increased in mortality rate [71–73]. These findings were similar to those for GCs therapy in the treatment of avian influenza [74].

GCs used in influenza patients incorporated with higher mortality. An important is that influenza virus is related to severe viral pneumonia, indeed diffuse alveolar damage may develop, high viral load reflects intense cytokine reactions, and systemic inflammation. It is suggested that GCs may play a role in increased replication of the virus. Certainly, the effects worsen the symptoms and disease that end up with mortality.

### 7.2. SARS

Since the outbreak in 2003, a respiratory disease, caused by coronavirus or well known as SARS, quickly spread around parts of the world; many studies have demonstrated empirical GCs therapy to treat SARS. At that time, because the urgency of the international outbreak did not allow time for efficacy studies, physicians in Canada and Hong Kong treated the earliest patients with intravenous ribavirin, broad-spectrum antiviral activity, and then followed by empirical GCs therapy and other treatment [75, 76].

Hien et al. confirmed that early hydrocortisone administration was initiated in <7 days of illness associated with significantly higher subsequent plasma viral load in second and third weeks; duration of viraemia may also be prolonged [71]. Ho et al. recommended that initial use of pulse methylprednisolone (≥500 mg/day) has more efficacious benefit such as reducing ICU admission, improving mechanical ventilation and mortality rate; it is also equally shown to be safer compared with low dosage [77]. Thus, low-dosage GCs also provided better prognosis in SARS patient's symptom and improved lung function [78–80]. In studies of SARS patients in Guangzhou, 121 of 152 critical patients (79.6%) received GCs at a mean daily dose of 133.5 ± 102.3 mg, which showed beneficial effect of GCs on mortality and shorter hospitalization days [76].

In October 2003, WHO established an International SARS Treatment Study Group in managing SARS and demonstrated optimal treatment options to deal with SARS. This systematic review reported summary effects of ribavirin, lopinavir, ritonavir (LPV/r), GCs, type I IFN, intravenous immunoglobulin (IVIG), or convalescent plasma in relation to (1) SARS-CoV replication inhibition in vitro, (2) mortality or morbidity in SARS patients, and (3) effects on ARDS in adult patients [81].

### 7.3. Pneumocystis jiroveci Pneumonia (PCP)

*Pneumocystis jiroveci* pneumonia (PCP) remains the most common high-risk opportunistic infection in patients associated with the human immunodeficiency virus (HIV). PCP contributed as one of the most common deadly infectious diseases, not only in HIV-infected patients but also in non-HIV patients with immunosuppressed condition.

The effectiveness of adjunctive GCs in reducing mortality and morbidity from *P. jirovecii* pneumonia in HIV patients has been demonstrated in a number of clinical trials. A meta-analysis by Briel et al. of six randomized clinical trials performed in the last decade demonstrated that adjunctive treatment with GCs was protective against *P. jirovecii* pneumonia. The risk ratios for overall mortality for adjunctive GCs therapy were 0.54 (95% confidence interval 0.38–0.79) at 1 month and 0.67 (0.49–0.93) at 3 months of follow-up. It was suggested that, in these patients, adjuvant GCs could prevent the need for mechanical ventilation and decrease mortality [82]. The combination trimethoprim-sulfamethoxazole together with GCs as primary agent for prophylaxis therapy was responded better result in patients with hypoxemia [83].

GCs show benefit in several HIV-related conditions and as adjunctive therapy in *Pneumocystis carinii* pneumonia. Existing data support the use of prednisone at 80 mg per day and tapered over 3 weeks in the management of severe P jirovecii pneumonia [78]. Tapering dose of GCs over 6–8 weeks is a reasonable option in T cell restoration. It has also been hypothesized that GCs treatment may benefit in HIV infection by reducing autoimmune components to CD4 depletion. However, there were potential side effects that may state in immunosuppression which are related to higher incidence of bacterial infections, herpes simplex and herpes zoster occurrence, and potential development of Kaposi's sarcoma [84]. It seems relevant whether risk factor of PJP is likely to be evolved in non-HIV settings where immunosuppressive agent therapy was administered. GCs use has preceded as one of the most frequent contributing agents [85–87].

The most frequently observed PJP underlying diseases were hematologic malignancies (54%), solid organ transplantation (17.4%), inflammatory disorders (13%), and solid cancer (10.8%) [81]. Patients with brain tumor who received GCs are also suspected to be in risk [88, 89]. Lack of benefit and increased risk factor of PJP demonstrated in those high dose GCs in patients with systemic lupus erythematosus patients [90]. Besides these negative results, the beneficial role of the application in non-HIV patients assumed that adjunctive high dose GCs (≥60 mg prednisone daily equivalent) improved recovery in cases of severe adult non-HIV PCP [91]. GCs either before, together, or later on with the specific antibiotic therapy may reduce respiratory complication, quickly resolve architectural distortions on HRCT chest in non-HIV immunocompromised patients [92].

Until there are more lines of evidences to direct practice, practitioners should continue to evaluate and recognize patients who are potentially to enroll treatment and prophylaxis and consider risk and benefit of adjunctive GCs in HIV and non-HIV patients.

## 8. Conclusion

The evidence study about efficacy of GCs provided significant reduction in levels of markers of systemic inflammation and duration of mechanical ventilation, ICU stay, and decrease mortality. GCs also have a wide range in diminishing the release of cytokines, such as those on plasma interleukin-6 levels, neutrophil counts, CRP levels in serum, and BAL.

Even though there were not enough lines evidence suggesting that GCs can be considered as adjunctive treatment due to its harmful effect and still unknown clinical outcome, we should consider that whether the respond to GCs is ineffective, effective, or harmful is influenced by drug dosage and duration of administration. We summarized that low to moderate dosage of GCs provides beneficial outcome. The potential benefit effect during GCs administration may be diminished if discontinuation of treatment is not preceded by slow tapering [39] and may lead to rebound inflammation noticed by increasing CRP value [30]. A better understanding of the interaction between systemic GCs and immune response is necessary before recommending their use in the treatment of severe pneumonia.

Until the results of new studies that are already in progress are published, it seems reasonable to think that some patients could benefit from the use of GCs, such as patients with severe pneumonia of certain etiologies, those with adrenal insufficiency, and those who develop septic shock with a poor response to the resuscitation maneuvers with liquids and perfusion of pressor amines. We suggested for future studies to pay attention to enroll a standardized treatment regimen, including timing, dosage, formulation, duration, and length of tapering.
